# Supplementary material for: Clinical characteristics and laboratory features of COVID-19 in high altitude areas: A retrospective cohort study
Source: PLoS One. 2021 May 18;16(5):e0249964. doi: 10.1371/journal.pone.0249964 (PMC8130933; doi:10.1371/journal.pone.0249964)
Supplement: S1 Table — (DOCX) [file pone.0249964.s001.docx]

S1 Table Adverse effects of the antiviral drugs

|  | Before Ribavirin treatment | After Ribavirin treatment | P value | Before Arbidol treatment | After Arbidol treatment | P value |
| --- | --- | --- | --- | --- | --- | --- |
| Red blood cell count (×10^9^/L) | 5.0(4.0-5.6) | 4.7(3.9-5.4) | 0.207 | 4.9(3.9-5.4) | 4.9(4.0-5.6) | 0.144 |
| White blood cell count (×10^9^/L) | 4.9(4.1-7.1) | 4.7(4.2-5.5) | 0.779 | 5.0(4.2-5.7) | 5.5(4.0-6.2) | 0.465 |
| Haemoglobin (g/L) | 158.0(128.8-175.8) | 156.0(124.5-170.0) | 0.292 | 159.0(126.3-170.3) | 161.0(131.8-173.8) | 0.197 |
| Platelet count (×10^9^/L) | 95.5(65.0-161.0) | 93.5(64.8-166.0) | 0.779 | 147.5(104.3-202.5) | 145.5(124.3-202.8) | 0.273 |
| Alanine aminotransferase (U/L) | 64.5(52.8-91.5) | 76.0(37.3-125.0) | 0.726 | 58.0(24.5-105.8) | 61.0(35.0-67.0) | 0.735 |
| Aspartate aminotransferase (U/L) | 46.5(43.5-67.8) | 44.5(29.0-118.5) | 0.401 | 44.5(20.0-69.8) | 53.0(25.0-67.0) | 0.611 |
| alkaline phosphatase (U/L) | 119.2(82.6-171.3) | 114.0(83.5-158.5) | 0.401 | 114.0(82.0-158.5) | 121.0(96.0-169.0) | 0.866 |
| y-Glutamyltranspeptidase (U/L) | 149.0(72.3-208.0) | 166.0(66.75-371.3) | 0.889 | 166.0(62.3-371.3) | 185.0(54.0-425.0) | 0.672 |
| Albumin (g/L) | 44.6(43.6-46.3) | 42.6(39.3-45.3) | 0.069 | 43.3(39.3-45.6) | 43.5(41.4-44.7) | 0.398 |
| Total bilirubin (μmol/L) | 6.4(4.6-9.8) | 11.3(6.9-14.6) | 0.017 | 8.1(5.9-14.6) | 8.7(7.5-19.2) | 0.043 |
