# Supplementary material for: Clinical characteristics and laboratory features of COVID-19 in high altitude areas: A retrospective cohort study
Source: PLoS One. 2021 May 18;16(5):e0249964. doi: 10.1371/journal.pone.0249964 (PMC8130933; doi:10.1371/journal.pone.0249964)
Supplement: S2 Table — (DOCX) [file pone.0249964.s002.docx]

S2 Table Patients’ outcomes between antiviral therapy group and non-antiviral therapy group

|  | Antiviral therapy(n=25) | Non-antiviral therapy(n=31) | P value |
| --- | --- | --- | --- |
| Nuclei acid tests negative for respiratory tract pathogen twice consecutively rate--No. (%)： | 14(56.0) | 24(77.4) | 0.088 |
| Days from nuclei acid tests positive to nuclei acid tests negative -median (IQR), days | 6.5(3.0-14.0) | 8.5(3.0-10.0) | 0.951 |

P values denoted the comparison between cases with antiviral therapy and cases without antiviral therapy.
