# Supplementary material for: Clinical characteristics and laboratory features of COVID-19 in high altitude areas: A retrospective cohort study
Source: PLoS One. 2021 May 18;16(5):e0249964. doi: 10.1371/journal.pone.0249964 (PMC8130933; doi:10.1371/journal.pone.0249964)
Supplement: Attachment — Submitted filename: Response to Reviewers.docx [file pone.0249964.s004.docx]

Response to Reviewers

Dear Editor:

On behalf of my co-authors, I am submitting the revised manuscript "Clinical characteristics and laboratory features of COVID-19 in high altitude areas: A retrospective cohort study" on PLOS ONE, a marked-up version and an unmarked version, along with additional Supporting Information files.

The manuscript has not been published before and is not being considered for publication elsewhere. All authors have read and approved the manuscript, contributed significantly to this work. We declare that there is no conflict of interest. Cost of reproducing color figures is waived because this article does not contain any pictures.

**For Journal Requirements:**

1. Please ensure that your manuscript meets PLOS ONE's style requirements, including those for file naming. The PLOS ONE style templates can be found at

https://journals.plos.org/plosone/s/file?id=wjVg/PLOSOne_formatting_sample_main_body.pdf and

<https://journals.plos.org/plosone/s/file?id=ba62/PLOSOne_formatting_sample_title_authors_affiliations.pdf>

**Response:** We confirm that our manuscript meets PLOS ONE's style requirements.

2. We note that you have indicated that data from this study are available upon request. PLOS only allows data to be available upon request if there are legal or ethical restrictions on sharing data publicly. For information on unacceptable data access restrictions, please see http://journals.plos.org/plosone/s/data-availability#loc-unacceptable-data-access-restrictions.

In your revised cover letter, please address the following prompts:

a) If there are ethical or legal restrictions on sharing a de-identified data set, please explain them in detail (e.g., data contain potentially identifying or sensitive patient information) and who has imposed them (e.g., an ethics committee). Please also provide contact information for a data access committee, ethics committee, or other institutional body to which data requests may be sent.

b) If there are no restrictions, please upload the minimal anonymized data set necessary to replicate your study findings as either Supporting Information files or to a stable, public repository and provide us with the relevant URLs, DOIs, or accession numbers. Please see http://www.bmj.com/content/340/bmj.c181.long for guidelines on how to de-identify and prepare clinical data for publication. For a list of acceptable repositories, please see http://journals.plos.org/plosone/s/data-availability#loc-recommended-repositories.

We will update your Data Availability statement on your behalf to reflect the information you provide.

**Response:** There are no restrictions of the data. We have uploaded the data as Supporting Information files.

3. Please review your reference list to ensure that it is complete and correct. If you have cited papers that have been retracted, please include the rationale for doing so in the manuscript text, or remove these references and replace them with relevant current references. **Any changes to the reference list should be mentioned in the rebuttal letter that accompanies your revised manuscript.** If you need to cite a retracted article, indicate the article’s retracted status in the References list and also include a citation and full reference for the retraction notice.

**Response:** We added the following references according to revision:

11.	Breevoort A, Carosso GA, Mostajo-Radji MA. High-altitude populations need special considerations for COVID-19. Nat Commun. 2020;11(1):3280. Epub 2020/07/03. doi: 10.1038/s41467-020-17131-6. PubMed PMID: 32612128.

17.	Fu L, Wang B, Yuan T, Chen X, Ao Y, Fitzpatrick T, et al. Clinical characteristics of coronavirus disease 2019 (COVID-19) in China: A systematic review and meta-analysis. J Infect. 2020;80(6):656-65. Epub 2020/04/14. doi: 10.1016/j.jinf.2020.03.041. PubMed PMID: 32283155; PubMed Central PMCID: PMCPMC7151416.

18.	Seclén SN, Nunez-Robles E, Yovera-Aldana M, Arias-Chumpitaz A. Incidence of COVID-19 infection and prevalence of diabetes, obesity and hypertension according to altitude in Peruvian population. Diabetes Res Clin Pract. 2020;169:108463. Epub 2020/09/25. doi: 10.1016/j.diabres.2020.108463. PubMed PMID: 32971150; PubMed Central PMCID: PMCPMC7505740.

20.	Qian GQ, Yang NB, Ding F, Ma AHY, Wang ZY, Shen YF, et al. Epidemiologic and clinical characteristics of 91 hospitalized patients with COVID-19 in Zhejiang, China: a retrospective, multi-centre case series. Qjm. 2020;113(7):474-81. Epub 2020/03/18. doi: 10.1093/qjmed/hcaa089. PubMed PMID: 32181807; PubMed Central PMCID: PMCPMC7184349.

We also exclude the following references according to revision:

22.	Wu PF, Li RZ, Zhang W, Hu HY, Wang W, Lin Y. Polycystic ovary syndrome is causally associated with estrogen receptor-positive instead of estrogen receptor-negative breast cancer: a Mendelian randomization study. American journal of obstetrics and gynecology. May 13 2020;doi:10.1016/j.ajog.2020.05.016

We updated the following references which were from medRxiv or bioRxiv:

24.	Liu J, Liu Y, Xiang P, Pu L, Xiong H, Li C, et al. Neutrophil-to-lymphocyte ratio predicts critical illness patients with 2019 coronavirus disease in the early stage. J Transl Med. 2020;18(1):206. Epub 2020/05/22. doi: 10.1186/s12967-020-02374-0. PubMed PMID: 32434518; PubMed Central PMCID: PMCPMC7237880.

26.	Huang H, Cai S, Li Y, Li Y, Fan Y, Li L, et al. Prognostic Factors for COVID-19 Pneumonia Progression to Severe Symptoms Based on Earlier Clinical Features: A Retrospective Analysis. Front Med (Lausanne). 2020;7:557453. Epub 2020/10/31. doi: 10.3389/fmed.2020.557453. PubMed PMID: 33123541; PubMed Central PMCID: PMCPMC7571455.

**Reviewers' Comments to the Author:**

Reviewer #1: The authors describe the clinical and laboratory characteristics of COVID-19 patients in high altitude areas of Sichuan province, China. According to the results present in this manuscript, the authors do not compare the patients in high and low altitude areas and only described a cohort study. Without the information of patients positive to COVID-19 in-plane area, it is difficult to correlate the symptoms and attribute some aspects with the geographic area. The results are clear and will be contributed to the knowledge of COVID-19 disease.
The manuscript must be reviewed at the grammatical and typographical levels (example: a lack of spaces between the words)
Although some aspects of these results must be improved, I recommend that this manuscript be revised.

**Response:** First of all, thank you for bring up this important issue. We are well aware of the limitations of our study which was that we were not able to compare the characteristics of COVID-19 patients in high and low altitude areas due to the inability to access the relevant data. Nonetheless, we tried our best to precisely describe all the clinical and laboratory features of COVID-19 in high altitude areas, allowing an adequate comparison with other previously published studies conducted in low altitude areas. Also, we described the similarities and differences in the clinical features of COVID-19 between high and low altitude areas in discussion part of the manuscript in detail.

Moreover, thanks for your suggestion of language editing of the manuscript. We have tried our best to polish the language in the revised manuscript. We believe the language has been improved in the updated version.

Sincerely Yours,

Xiaohong Zhang

Deapartment of Emergency, Sichuan Provincial People’s Hospital

No.32 West Section 2, 1st Ring Road, Qingyang District, Chengdu, 610072, Sichuan Province, P. R. China

Tel/Fax: 18981838173

E-mail: 2653099978@qq.com
